# Supplementary material for: Evaluating Complexity of Fetal MEG Signals: A Comparison of Different Metrics and Their Applicability
Source: Front Syst Neurosci. 2019 May 27;13:23. doi: 10.3389/fnsys.2019.00023 (PMC6546028; doi:10.3389/fnsys.2019.00023)
Supplement: Supplementary file 1 [file Table_1.DOCX]

Supplementary Material

**Evaluating Complexity of Fetal MEG Signals: A Comparison of Different Metrics and Their Applicability**

Julia Moser^1*^, Siouar Bensaid^2^, Eleni Kroupi^3^, Franziska Schleger^1^, Fabrice Wendling^2^, Giulio Ruffini^3^, Hubert Preißl^1^

*** Correspondence:** Julia Moser: julia.moser@student.uni-tuebingen.de

# Scale-free approaches

A given process $X(t)$ is said statistically self-similar if its statistical properties are invariant after rescaling and time dilating

$X(t)\underset{\Leftrightarrow}{d}s^{-H}X(st)$ (1)

where $\underset{\Leftrightarrow}{d}$ denotes statistical equivalence and $s$ is a scalar scaling factor. This self-similarity implies a power-law behavior of statistical moments so that the *q*^th^ statistical moment of the process$E\left| X\left( t \right) \right|^{q}$is expressed as

$E\left| X\left( t \right) \right|^{q}\propto\left| t \right|^{qH}E\left| X\left( 1 \right) \right|^{q}$ (2)

where *H* refers to the *Hurst* exponent. In the multifractality scheme, $X(t)$ is no longer characterized by one exponent, but rather several exponents *h*, called *Hölder* exponents, forming the multifractality spectrum$D(h)$ whose maximum coincides with the *Hurst* exponent. The *q*^th^ statistical moment is then expressed as

$E\left| X\left( t \right) \right|^{q}\propto\left| t \right|^{\zeta(q)}E\left| X\left( 1 \right) \right|^{q}$ (3)

where $\zeta(q)$ is a scaling polynomial (concave) function. The latter is related to the multifractality spectrum$D(h)$ (a.k.a. singularity spectrum) via the Legendre transform

$D\left( h \right)= \min_{q\neq0} (1+qh-\zeta(q))$ (4)

Multifractal analysis amounts to analyze the signal $X(t)$ across different scales $s$.There are two practical well-known approaches to measure fractality in a process (if it exists).

## Multifractal detrended fluctuation analysis

Given a time series$X(t)$, the fluctuating function ${F_{q}(s)}^{q}$is defined as

${F_{q}(s)}^{q}$ = $\frac{1}{N}\sum_{i=1}^{N} \sigma_{i,s}^{q}$ (5)

where *s* and *N* represent, the scale and the number of segments of length, respectively. $\sigma_{i,s}$ is the standard deviation of the detrended signal in segment *i* at scale *s*. When $X\left( t \right)$ is multifractal, the fluctuating function ${F_{q}(s)}^{q}$presents a power-law scaling behavior

${F_{q}(s)}^{q}\sim s^{H(q)}$ (6)

where *H*(*q*) denotes the generalized *Hurst* exponent (*q*-dependent). The latter is related to the scaling function by$\zeta\left( q \right)=qH\left( q \right)-1$ and to the *Hölder* exponent by$h\left( q \right)=qH'\left( q \right)+H\left( q \right)$. The Multifractal detrended fluctuation analysis (MFDFA) computes $h(q)$ by linearly regressing $\log{F_{q}\left( s \right)}^{q}$ versus the scale log(*s*) for each value of *q*. After few computational steps (detailed in (Kantelhardt 2002)), the multifractal spectrum can be calculated, without using the Legendre transform, as

$D\left( h \right)=q\left( h(q)-H\left( q \right) \right)+1$ (7)

The *Hurst* exponent *H* and the spectral width *M* correspond to the *h* maximizing $D\left( h \right)$and the width of$D\left( h \right)$, respectively.

In the monofractal case, $\zeta(q)$ is reduced to a linear function of *q* so that$\zeta\left( q \right)=qH-1$, where *H* is the *Hurst* exponent.

## Wavelet Leader-based multifractal approach

Given a time series$X(t)$ and for a fixed analysis scale$2^{j}$, the structure function $S_{X}\left( j,q \right)$is defined as

$S_{X}\left( j,q \right)=\frac{1}{n_{j}}\sum_{k=1}^{n_{j}} \left| L_{X}(j,k) \right|^{q}$ (8)

where $L_{X}(j,k)$ is the wavelet leaders coefficient at scale *j* and time *k* and $n_{j}$ is the number of $L_{X}(j,k)$ available at scale $2^{j}$. When $X(t)$ is a fractal process, the structure function shows a power-law behavior

$S_{X}\left( j,q \right)\propto2^{j\zeta(q)}$ (9)

By noting that $S_{X}\left( j,q \right)$ is a sample mean estimator of $\mathbb{E}\left[ {L_{X}(j,.)}^{q} \right]$ and using the standard generating function expansion, the following relation can be established

$\ln\mathbb{E}\left[ e^{q\ln L_{X}\left( j, \right)} \right]=\sum_{p=1}^{\infty} C^{L}\left( j,p \right){(q}^{p}/p!)$ (10)

where $C^{L}\left( j,p \right)$stands for the cumulants of$\ln L_{X}(j,.)$ of order $p\geq1$. Combining (9) and (10) compels that these cumulants satisfy the following form

$C^{L}\left( j,p \right)$=$c_{0,p}+c_{p}\ln2^{j}$ $\forall p\geq1$ (11)

which consequently yields$\zeta\left( q \right)=\sum_{p=1}^{\infty} c_{p}\frac{q^{p}}{p!}$. The characterization of $\zeta\left( q \right)$ (consequently$D\left( h \right)$) amounts to calculating the log-cumulants$c_{p}$. In terms of interpretations, the log-cumulants $c_{1}$and $c_{2}$ characterize the maximum and width of the multifractal spectrum$D\left( h \right)$. In the WLBMF method, the log-cumulants are estimated by linearly regressing the estimate of cumulants $\hat{C}^{L}\left( j,p \right)$ versus $\ln2^{j}$ in the analyzed scales range $[j_{1},j_{2}]$

${\hat{\boldsymbol{c}}}_{\boldsymbol{p}}\boldsymbol{=} \mathbf{log}_{\boldsymbol{2}} \boldsymbol{e} \sum_{\boldsymbol{j=}\boldsymbol{j}_{\boldsymbol{1}}}^{\boldsymbol{j}_{\boldsymbol{2}}} \boldsymbol{w}_{\boldsymbol{j}}{\hat{\boldsymbol{C}}}^{\boldsymbol{L}}\left( \boldsymbol{j}\boldsymbol{,}\boldsymbol{p} \right)$ (12)

The estimates $\hat{C}^{L}\left( j,p \right)$ are calculated using the standard methods of cumulant estimators. For more details, see (Wendt, Abry et al. 2007).

**References**

Kantelhardt, J. W. Z., Stephan A.; Koscielny-Bunde, Eva; Havlin, Shlomo; Bunde, Armin; Stanley, H. Eugene (2002). "Multifractal Detrended Fluctuation Analysis of Nonstationary Time Series." Physica A: Statistical Mechanics and its Applications **316**(1): 87-114.

Wendt, H., P. Abry and S. Jaffard (2007). "Bootstrap for Empirical Multifractal Analysis." IEEE Signal Processing Magazine **24**(4): 38-48.
